# Forest Goers and Multidrug-Resistant Malaria in Cambodia: An Ethnographic Study

**DOI:** 10.4269/ajtmh.18-0662

**Published:** 2019-03-11

**Authors:** Melanie Bannister-Tyrrell, Charlotte Gryseels, Suon Sokha, Lim Dara, Noan Sereiboth, Nicola James, Boukheng Thavrin, Po Ly, Kheang Soy Ty, Koen Peeters Grietens, Siv Sovannaroth, Shunmay Yeung

**Affiliations:** 1 Institute of Tropical Medicine, Antwerp, Belgium;; 2 Center for Health and Social Development, Phnom Penh, Cambodia;; 3 London School of Hygiene and Tropical Medicine, London, United Kingdom;; 4 National Center for Parasitology, Entomology and Malaria Control, Phnom Penh, Cambodia

## Abstract

Multidrug-resistant *Plasmodium falciparum* malaria on the Cambodia–Thailand border is associated with working in forested areas. Beyond broad recognition of “forest-going” as a risk factor for malaria, little is known about different forest-going populations in this region. In Oddar Meanchey Province in northwestern Cambodia, qualitative ethnographic research was conducted to gain an in-depth understanding of how different populations, mobility and livelihood patterns, and activities within the forest intersect with potentiate malaria risk and impact on the effectiveness of malaria control and elimination strategies. We found that most forest-going in this area is associated with obtaining precious woods, particularly Siamese rosewood. In the past, at-risk populations included large groups of temporary migrants. As timber supplies have declined, so have these large migrant groups. However, groups of local residents continue to go to the forest and are staying for longer. Most forest-goers had experienced multiple episodes of malaria and were well informed about malaria risk. However, economic realities mean that local residents continue to pursue forest-based livelihoods. Severe constraints of available vector control methods mean that forest-goers have limited capacity to prevent vector exposure. As forest-goers access the forest using many different entry and exit points, border screening and treatment interventions will not be feasible. Once in the forest, groups often converge in the same areas; therefore, interventions targeting the mosquito population may have a potential role. Ultimately, a multisectoral approach as well as innovative and flexible malaria control strategies will be required if malaria elimination efforts are to be successful.

## INTRODUCTION

Malaria, caused by the infection of red blood cells by *Plasmodium* parasites and transmitted by the bite of female *Anopheles* mosquitoes, occurs throughout much of the world’s tropical and subtropical regions. At global, regional, and local scales, there is substantial variation in malaria risk, in terms of population at risk^1^; intensity of transmission; climatic, geographic and temporal variations; and variations in human, vector, and parasite ecology.^2^ Forested regions have been consistently identified as having distinct malaria transmission characteristics, and the term “forest malaria” describes diverse epidemiological settings in the Amazon, Central African, Western Pacific, and Southeast Asian regions, in which malaria transmission occurs and is associated with residence and population movements in forest or forest-fringe ecosystems where competent vector populations reside.^3–5^ Increased risk of malaria in forested regions has been related to the presence and behavior of multiple forest-dwelling vector species, climatic and topographic conditions conducive to vector breeding, zoonotic malaria infections, deforestation and land use change, predominance of impoverished and/or ethnic minority populations, migration and livelihood patterns, poor health infrastructure and access, open housing construction, and numerous other factors.^3^

The incidence of malaria in Southeast Asia declined overall by 54% between 2000 and 2015, a period coinciding with socioeconomic development and intensified malaria control efforts,^6^ but also with deforestation (1.45 million hectares per year between 2000 and 2010^7^). Malaria in Southeast Asia, including Cambodia, is now largely confined to populations living in the remaining forested regions, mostly in remote areas and adjoining international borders.^8^ In Cambodia, distinct populations have been shown to be at risk of forest malaria, including forest-fringe inhabitants^9^; mobile and migrant people who spend extended periods of time staying and working in the forest, for example, gem miners, loggers, or soldiers^10,11^; and ethnic minorities practicing subsistence slash and burn agriculture in forest farms and fields.^12–14^ Forested international border regions receive particular attention, with the preponderance of forest-based activities combined with poor access to health care contributing to higher malaria prevalence than surrounding regions.^15^ For economic reason, there are high-volume internal mobility and cross-border activities between Cambodia and Thailand, where the malaria burden is still present. Depending on the setting, the demographic groups at risk of contact with forest-dwelling vectors, and hence malaria infection, vary according to how people interact with different forested landscapes. In settings where transmission occurs in forested areas away from village locations, occupational profiles are strongly linked to malaria risk and adult men constitute most of the malaria cases.^10^

However, static and broad risk classifications based on dominant landscape or population type do not engage with how dynamic human–forest interactions lead to a variable and fluctuating local malaria, despite recognition that malaria epidemiology varies according to the local, social, and environmental contexts.^5,16,17^ Extensive focus on forest-going mobile populations as a “risk group”^18^ stands in contrast to limited efforts to elucidate how different populations, mobility patterns, and landscapes interact to potentiate and maintain forest malaria transmission.

Despite low incidence rates, remaining forest malaria foci on the Thailand/Cambodia border constitute the epicenter for the emergence of *Plasmodium falciparum* drug resistance,^19,20^ which makes the region an international priority for accelerated malaria elimination.^21^ The Cambodian government aims to achieve elimination of *P. falciparum* malaria and malaria deaths by 2025.^22^ Qualitative ethnographic research was conducted in this region to gain an in-depth understanding of forest activities among individuals at risk of malaria infection. This article focuses on characterizing and distinguishing different types of “forest-goers” and forest activities in relation to their malaria risk and discussing the implications for malaria elimination in this region.

## MATERIALS AND METHODS

### Study setting.

**Figure 1.**
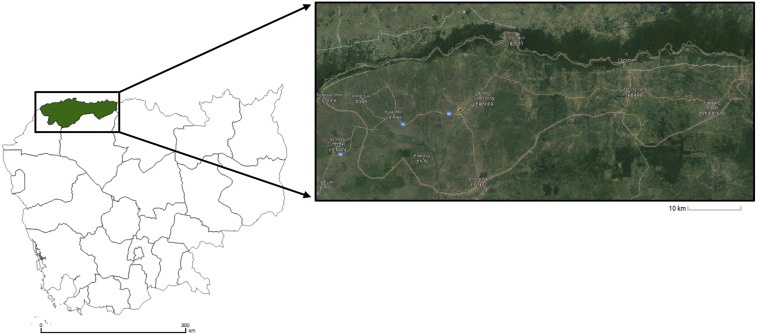
Map of Cambodia showing satellite map of Oddar Meanchey Province inset. Imagery © Landsat/Copernicus. Map data © 2017 Google. This figure appears in color at www.ajtmh.org.

The study was conducted in Oddar Meanchey Province, in northwestern Cambodia, which borders Thailand (Figure 1). The population of approximately 231,387 (2013 estimate^23^) comprises recent migrants and longer term residents, with most villages and towns clustered in the eastern half of the province. Although once one of the most heavily forested and remote areas in Cambodia, it is now widely deforested. Deforestation occurred in two phases: some land clearing occurred during Khmer Rouge era, when the region became the last stronghold and de facto capital of the Khmer Rouge until their expulsion in 1997. Since then, rapid agricultural expansion, economic land concessions, land clearing and logging, and population growth driven by internal migration from other Cambodian provinces have drastically reduced the total forest cover in the province,^24^ with densely forested areas now largely confined to the Dangrek mountain range which forms the border with Thailand (see inset satellite map in Figure 1).

Malaria epidemiology in Oddar Meanchey is highly focal and unstable, with low and declining prevalence overall (0.8% infection prevalence by polymerase chain reaction in November 2015 survey), but prevalence is higher in some villages and sporadic outbreaks occur (unpublished data). Both *P. falciparum* and *Plasmodium vivax* malaria occur (0.4% *P. falciparum*, 0.3% *P. vivax*, and 0.1% mixed infection in the November 2015 survey). Prevalence of parasite drug resistance to the first-line artemisinin-based combination therapy of dihydroartemisinin and piperaquine exceeded 50% in 2015,^25^ necessitating a policy switch to the previous first-line combination of artesunate–mefloquine.

### Study design.

Ethnographic research was conducted as part of the Proactive Case Detection and Community Participation for Malaria Elimination Study (PACES), a cluster-randomized trial of the impact of proactive screening and treatment for asymptomatic malaria infections, delivered through village malaria workers (VMWs). The screening algorithm for the trial included “forest-going” as a key risk behavior for *P. falciparum* infection, in which “forest-going” was defined as having spent at least one night in the forest (away from the village) in the past month. The overall objectives of the social science component of PACES were to gain an in-depth understanding of forest-going activities to inform malaria elimination activities and to explore the acceptability and feasibility of the PACES intervention.

The ethnography research team comprised the first author and three Cambodian researchers all trained and experienced in ethnographic research methods. Data were collected in two phases: a preliminary exploratory phase from November to December 2015, after a baseline survey but before randomization and the commencement of the trial, and from May 2016 to April 2017 during the onset and continuation of the trial. The first phase comprised background ethnographic research focused on understanding the general social context in the study area, characteristics of forest-goers, and activities undertaken in the forest, and the second phase focused on understanding conditions for and trajectories of forest-based livelihoods, as well as perceptions and experiences of malaria among the forest-going and forest-fringe–dwelling populations.

### Sampling.

Theoretical sampling was used to select informants, which implies purposive and gradual selection of participants in accordance with emerging theory and efforts to maximize variation in the sample. Villages were purposively selected based on malaria prevalence, proximity to the forest, and proportion of residents who knew the VMW in their village as reported in the baseline survey. The sample included 1) key position holders, such as VMWs, staff at public health facilities, private health-care providers, and representatives of local sociopolitical power including village chiefs and vice chiefs, as well as forest-going group leaders; 2) forest goers; and 3) other members of the community, including non–forest-goers and families of forest-goers. Forest goers were selected and classified based on the following characteristics: migration status (including local residents and recently arrived migrants), age (including adolescent and older forest goers), gender, and type of forest activity.

### Data collection and analysis.

During the fieldwork, qualitative data collection and analysis were concurrent and iterative. Interviews, informal conversations, and observations were conducted in 24 villages in the eastern half of Oddar Meanchey Province, with a focus on the highest prevalence villages along the border with Thailand. In-depth semi-structured and open-ended interviews (*n* = 44) were audio-recorded when possible. For interviews jointly conducted by the first author and research assistants, questions posed by the first author in English were translated into Khmer by the research assistant, who then briefly summarized the participant’s response(s) in English at appropriate times in the interview. Interviews conducted independently by research assistants were in Khmer. Audio-recorded interviews were transcribed verbatim by Cambodian research assistants with the aid of F4 transcription software (f4transcript©, Marburg, Germany), with pauses, interjections, and laughter indicated in the transcripts. The transcription software was used to slow down, pause, and resume transcriptions, but no automatic speech recognition was used. English translations were then added line by line to the Khmer transcript, which allowed for error checking and review of either the transcription or translation. Transcription and translation into English were performed by the same Cambodian research assistants who conducted the interviews. Informal conversations (*n* = 44) that were summarized but not audio-recorded were preferred by participants in some instances, especially when discussing sensitive topics about forest activities. Numerous informal group conversations were conducted, often incidentally when research participants were joined by family members or friends over the course of an interview. Participant observations of intervention activities and monthly meetings for public health-care staff at health centers (*n* = 33) were also recorded, and observations about the general social context in the study villages were regularly discussed and recorded in field notes. Preliminary findings were regularly reviewed, question guides were revised, and additional topics and target groups were identified. Data collection continued until saturation was reached.

Data were imported into NVivo (Version 11, © QSR International) for data management and analysis. An open inductive coding approach was initially used to assign tentative codes, with themes emerging and being refined during this process. A log was maintained from the beginning of the coding process to record any pertinent observations, early impressions, and potential lines of enquiry. After preliminary coding was completed, existing codes were reviewed and revised, and incorporated into a thematic coding tree, which was applied to all data. Abductive analysis was used throughout to refine and analyze themes grounded in the data.^26^

### Ethics.

The study protocols for the ethnographic subcomponent of PACES and the overall PACES study were approved by the National Ethics Committee for Health Research, Cambodia, and the Institutional Review Boards of the London School of Hygiene and Tropical Medicine, United Kingdom, and the Institute of Tropical Medicine, Belgium. The ethnographic study was conducted in accordance with the Code of Ethics of the American Anthropological Association and the Declaration of Helsinki. All research participants provided verbal consent to participate after being informed of the objectives of the ethnographic research and were free to withdraw at any time. Consent processes for the ethnographic research were separate from and additional to the PACES study, and raw data or any identifying information were available to the ethnography research team only.

## RESULTS

In Oddar Meanchey Province, much of the population is engaged in forest-based livelihoods, including 1) logging and related activities in the forest; 2) agricultural and other work in forest-fringe areas, where farms and plantations are located, as well as carpentry workshops, and where land clearing continues to make way for agriculture, or new or expanded settlements; and 3) residence in extant forest-fringe villages and settlements.

The ethnographic researchers, the PACES trial team, and local the VMWs observed that most of the malaria cases were people who had spent evenings or nights in the forest for logging-related activities; therefore, this population became the focus for the research.

### Types of forest-goers.

#### Local residents.

A range of professional profiles were apparent among forest-goers.† Loggers make up most of the people involved, most of whom are men aged 20 to 30 years, reflecting the strenuous work requirements, although there were also some adolescent boys and older men. In some villages, a majority of resident men working were active forest-goers, whereas in other villages, it was relatively few. Although a large majority of forest-goers are men, a few adult women also go logging with other women and/or male relatives, especially widows or women whose husbands are unable to work.

In short, all men living in this village go to forest. We depend on it for living.

(In-depth interview with adult forest goer, forest fringe village)

#### Temporary migrants.

Temporary migrants usually stay in small rented huts that line the main road of some of the villages or are hosted by the team leader or middleman who has organized their logging trips. Informants reported that rosewood stocks substantially declined in 2016–2017, and consequently, fewer migrants arrived in the region compared with previous years. In addition, after recent crackdowns on illegal logging, some migrants consider logging to be too dangerous to continue. Many migrants who resided in Oddar Meanchey have returned to their home provinces, or have moved to Thailand to seek employment in factories or on construction sites.

#### Additional key professional roles.

Apart from forest-goers who are engaged directly in logging, there were several other key roles that had varying levels of exposure to forest malaria themselves but that provide a potential point of contact for reaching teams of forest-goers. This includes “team leaders” and “middlemen” who broker arrangements locally and negotiate the price with buyers. Team leaders are usually local adult men with previous logging experience and excellent knowledge of the local mountain terrain. Typically, only recently arrived and short-term migrants go logging with a team leader. With the decline in arrivals of mobile people in the area, the proportion of forest-going groups led by a team leader is also declining. Unlike team leaders, middlemen do not go to the forest themselves, and are therefore less at risk themselves of getting malaria.

#### Other roles.

Additional roles in the timber trade who are at risk of malaria include brokers, interpreters, “forest hosts,” transporters, and carpenters. The brokers and interpreters accompany large forest-going groups of mostly short-term migrants led by team leaders. “Forest hosts” are individuals who have houses within the forest where forest-goers stay overnight and store their timber during multiday logging expeditions, and “transporters,” are individuals who do not go logging but make multiple trips to and from the forest to transport timber down the mountain. In addition, many carpenters have workshops in the Dangrek Mountains, at the foothills, or in the forest-fringe villages, where they buy timber and produce furniture. The furniture is sold to middlemen who travel to their workshops or to other direct buyers. Forest workshops reduce costs of transporting timber purchased on the mountain and enable carpenters to additionally work as loggers, all the while maintaining family houses in villages several kilometers from the forest fringe.

### Patterns of forest exposure.

#### Duration of exposure in the forest.

Most forest-going groups (including loggers, team leaders, brokers/interpreters, and transporters) work in the evening and through the night, coinciding with vector biting hours.^27^ Most forest-going local residents report going to the forest around 10 times per month, although larger forest-going groups may only make arrangements to go once or twice per month, while staying several nights to 2 weeks at a time. Some individuals with other income sources or occupations (including students) go less regularly, on an as-needs basis to supplement their own or their family income.

Time spent in the forest varies with both the distance traveled within the forest and the distance back to the individuals’ home villages. Travel times on the way to the forest are much faster than those on return, when the forest-goers reportedly carry up to 60 kilograms of timber. A 6- to 7-km walk may take just over 2 hours outbound but takes close to one day on the return journey. Previously, most forest-going groups spent one or two nights in the forest, leaving their villages in the late afternoon to reach the forest after dark. However, as rosewood has become more scarce, longer trips of up to 2 weeks are increasingly common. Women tend not to travel as deep into the forest as many men and are more inclined to make day return trips, and accordingly tend to spend less time in the forest. Those who transport timber down the mountain typically stay at least one night in the forest.

#### Pathways and intersections in the forest.

Some experienced forest workers travel in small groups of only two or three, but most go in larger groups of four to 10 people, typically trusted family members or neighbors. Recent or short-term migrants go logging in larger groups of 20 to 40 individuals, a decline from that of previous years when groups of 60 or 80 individuals, mostly migrants, would join logging trips. Locals usually go logging in groups with the same people each time, but rarely interact with other groups. They are often aware of other groups that go to the forest, but tend not to know them by name, especially if not from the same village.

Forest workers departing from their villages travel between two and 15 km by motorbike or on foot to reach the base of the Dangrek mountains. There are multiple routes leading to “entrances” or “gates,” which are informal border crossings marked by stone formations or proximity to a local landmark (including, e.g., a local house or pagoda). From these entrances, rough narrow walking tracks lead into the forest. Some of the more popular entrances are reportedly used by 100 or more individuals from several different groups over the course of an evening. However, there are numerous entry points to the forest, with many forest-fringe villages having up to six entrances. Some entrances are used by residents of one village only, whereas more frequented entrances are used by multiple groups, who may not know each other and come from different villages. Different groups may pass through the same entrance, but spread out in different directions in the forest, with some groups traveling as far as 15 km within the forest. Conversely, some groups take separate entrances, but meet and work in the same “block” within the forest. Regardless of the entrance used, different logging groups may be often working and resting within relatively close proximity to other groups. Informants estimate that there may be up to 60 forest-goers within one square kilometer at any given time when in the forest at night.

#### Prevention of vector exposure in the forest.

The national malaria control program has widely distributed insecticide-treated nets, both bed nets and hammock nets, in an effort to prevent malaria in high-risk communities. However, forest-goers reported that they seldom take either insecticide-treated bed nets or hammock nets on their trips into the forest. Most of them work through the night, and if they do rest, it is only for 1 or 2 hours. The nets are considered cumbersome and unnecessary, given the lack of night rest. If they do sleep, they either rest on the ground or in low-slung hammocks without nets.

Some forest-goers report to irregularly use mosquito coils and personal topical repellents that can be purchased locally. However, they commonly fear that the scent of coils or repellents will attract unwanted attention from forest rangers, limiting their use.

### Malaria risk perception and management.

Most informants referred to mosquito bites as the cause of malaria infections and associated the forest most strongly with malaria risk. However, forest-fringe village residents also perceived that people who do not go to the forest can also have malaria. Many were able to distinguish between *P. falciparum* and *P. vivax* malaria, according to the results of rapid diagnostic tests, and some respondents used the local term specifically for *Anopheles* mosquitoes (“Mous Daek Koul Nhi”) when describing the source of malaria transmission. Informants also commonly referred to other causes of malaria, including consuming unclean food and water, and exhaustion from strenuous work.

Interviewer: “Normally, do you know if malaria happens to mostly forest goers or villagers”?

Informant: “Not only for forest goers, malaria also happens to people in village because it is transmitted by mosquitos from one person to another person. Female anopheles (Mous Daek Koul Nhi) transmit malaria from one person to another person. Another reason is that we eat unhygienic, unclean food. That’s why it can be transmitted to them. Not only forest goers have malaria”

(In-depth interview with adult male forest goer)

The forest in general is recognized as a place where people can fall ill with fevers, and this is more specifically perceived to be related to the inadequate food and clean water intake, use of stimulant drugs, lack of bed or hammock net use, and strenuous work efforts associated with forest work. Malaria is commonly recognized by its symptoms (fevers and chills) and distinguished from other febrile illnesses, such as typhoid fever. For those who developed fever while working in the forest, their first recourse was typically to take paracetamol and then continue working if this alleviated their discomfort, possibly visiting a health provider after returning to the village. Those who fell more seriously ill or whose fevers progressed while working in the forest usually reported to return to their home village, but may also stop first at border towns and villages to seek treatment before continuing to their home village. These treatment stopovers usually entail the purchase of intravenous fluid infusions, a popular adjunctive therapy for fever in Cambodian private and public medical facilities.

Nevertheless, for most people, malaria episodes were frequent but otherwise unremarkable. It was common knowledge that malaria is diagnosed via a blood test, although people with suspected malaria seek health care at a range of practitioners, including VMWs, public health centers, private providers, and formal and informal pharmacies, as commonly observed in Cambodia.^28,29^ People with a history of repeated episodes of malaria claimed to recognize malaria by its symptoms alone, without need for a formal diagnosis.

Interviewer: “Have you ever had malaria?”

Informant: “Oh, [it] could not be counted if talking about me and malaria [laughing]. Malaria always happens…many times, yes, sometimes two times per month.”

(In-depth interview with adult male farmer and forest goer)

### The socioeconomic realities that support persistent malaria risk.

Longer term residents had better access to land when they moved to the area than more recent migrants. More recent arrivals, however, commonly own plots of land that are too small for income from farming to sustain their families. With fluctuations and declines in the rice and cassava prices over recent years, returns generated from annual harvests are often paltry and may be exceeded by the cost of inputs including fertilizer, fuel for small tractors, and transportation. Some farmers, therefore, supplement their income with trips to the forest specifically to earn money to purchase fertilizer or other farming supplies.

“Yes, I took the money I earned from the wood business, $100 or $50 per time. I went there [the forest] because we need fertilizer to use in the farm every 3 to 4 months.”

(In-depth interview with elder male farmer and forest goer)

In addition, some agricultural work is of relatively low intensity. For example, cassava farming is intensive during the harvest period, but otherwise requires limited time input throughout the year, freeing farmers to go logging as means to improve their earnings and have access to a cash flow between harvests. Both locals and migrants also work as hired laborers during intensive agricultural periods. Only a small proportion of village residents earn regular salaries. However, the low wages paid lead many to supplement their income with logging, land clearing, or plantation work, which reflects the ubiquity of local residents engaging in forest-based activities regularly or intermittently as part of flexible livelihood strategies.

Even as rosewood becomes scarce, local villagers report that they will continue going to the forest as long as there is demand for wood. As one local resident expressed,

It depends on the buyer. For example, if they will buy a thumb-sized stump, we can find it for them.

(In-depth interview with adult male farmer and occasional forest-goer)

The prospect of a “big win” further drives the continuation of working with rosewood.

It is not easy to change because we do not have anything to do besides looking for Kranhoung. Yes, for women they work under the sun [as hired workers] and it is very hot, they can earn just 20,000 riel [$5 USD]. If they go to carry [Kranhoung] one time, they can earn from 30,000 riel [$7.5 USD] to 100,000 riel [$25 USD]. If we can dig and get big, [we] can get 500,000 [$125 USD] to 600,000 riel [$150 USD] sometimes 200,000 riel [$50 USD].

(In-depth interview with adult male forest-goer)

Until recently, some forest goers could reportedly earn several thousands of dollars per year and were able to construct large houses, purchase cars and motorbikes, and purchase farm land in Oddar Meanchey or in their home provinces. The decline in availability and quality of rosewood has reduced the flow of migrants to the region, but many longer term and permanent local residents continue to go to the forest. Compared with previous years, logging now generates just enough income after costs to support households at little more than subsistence level.

If we don’t go to look for Kranhoung then we earn nothing. Sometimes we could earn 150,000 riel [$37.5 USD]. So how much could we share among members? [We have to] spend on diesel fuel, spend on road security [guards/officers], so we are left with around 20,000 to 30,000 riel per time [$5-6 USD], and that’s for one day. Tomorrow if we don’t go then we would earn nothing.

(In-depth interview with adult male forest goer)

Many locals have considerable debts, including forest-goers who took out loans from microfinance organizations.

“If we take loans to buy [expensive] goods, then when we get money from looking for Kranhoung, we use this money to pay back the loan. I owed [the lender] a thousand dollars but I can get money from Kranhoung [to pay it back].

(In-depth interview with adult male forest goer)

### Health risks for forest goers.

Logging exerts a high physical toll, with forest-goers routinely carrying dozens of kilos of wood on their backs and working throughout the night. While in the forest, workers report drinking unclean water and having limited or no food, making exhaustion inevitable. To counter these challenges, the use of stimulant drugs, such as methamphetamine, is common and often as a substitute for taking food supplies to the forest.

## DISCUSSION

### Conceptual model for drivers of local forest malaria epidemiology.

Extensive focus in the literature on “forest-going” and “mobile populations” as a “risk group”^18^ stands in contrast to limited efforts to elucidate how different populations, mobility patterns, and landscapes interact to potentiate and maintain forest malaria transmission. Static and broad risk classifications based on dominant landscape or population type have not engaged with how dynamic human–forest interactions lead to a variable and fluctuating local malaria, despite recognition that malaria epidemiology is heterogeneous and varies according to the local social and environmental context.^5,16,17,30^ For example, in this study, although most of the forest-goers were adult males, there were also female forest-goers, who are an under-recognized risk group for forest malaria in this setting.

**Figure 2.**
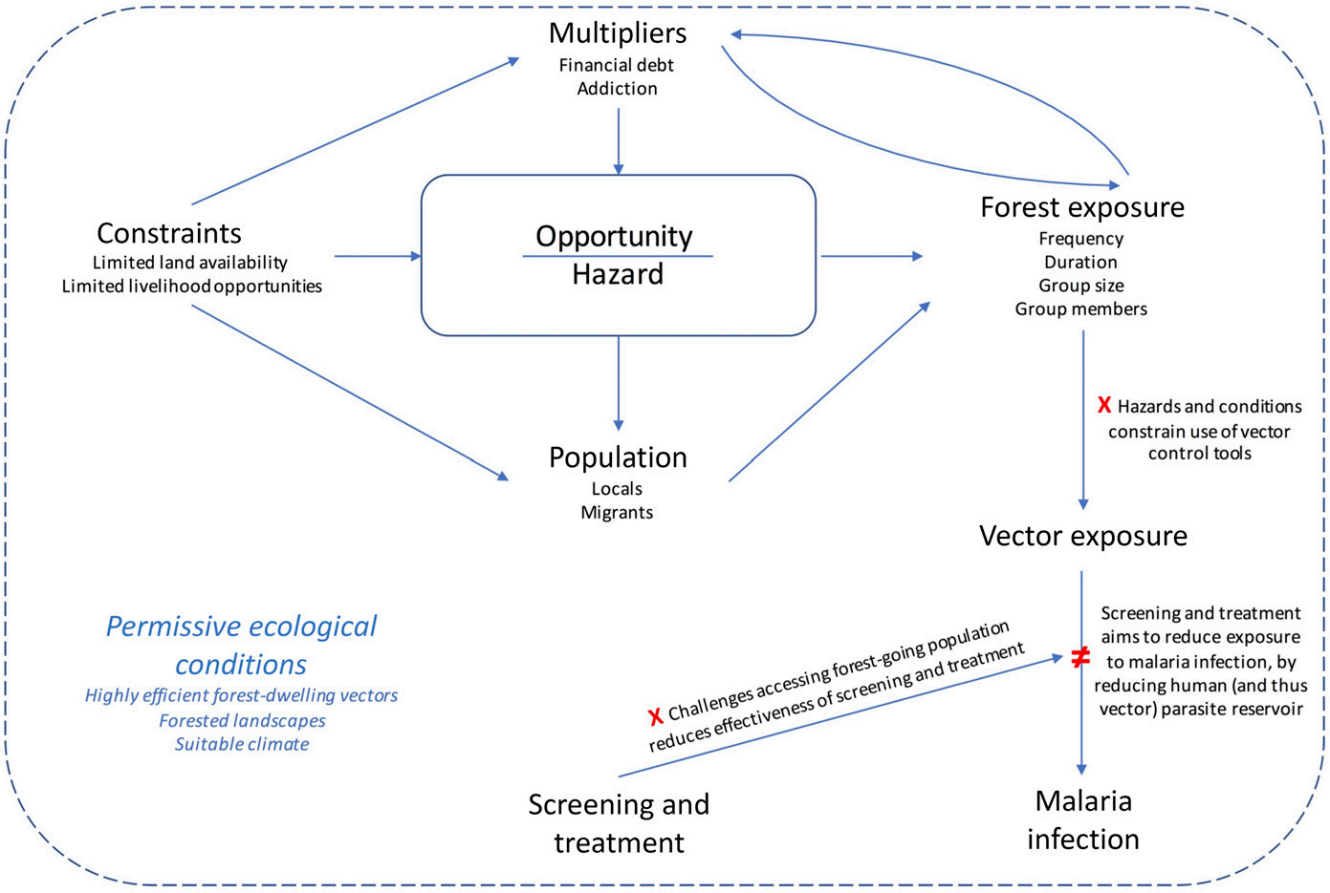
Conceptual model for determinants of local malaria epidemiology among forest-goers participating in the luxury timber trade in Oddar Meanchey Province, Cambodia. This figure appears in color at www.ajtmh.org.

Based on the findings of this study, we hypothesize that intra-annual and interannual variations in malaria incidence among forest exposed are driven primarily by fluctuations in population movements to, and activities in, the forest, which is determined by whether the profits that can be made in the timber trade outweigh the various hazards of working in the forest (Figure 2). Village malaria workers perceived a correlation between the number of malaria cases and the frequency of forest-going. The decline in malaria cases in 2016 compared with that in previous years was seen to be due to the downturn in new migrant arrivals. The remaining local loggers are now spending more nights in the forest, increasing their duration of exposure in the forest and making it even more difficult for them to reach appropriate health-care facilities should they become sick or injured, or for them to be reached as part of within-forest malaria elimination efforts.

It is clear that multiple forest-going groups congregate over relatively small areas in the forest, which suggests there may be “hotspots”^31^ within the forest where human and vector populations converge at sufficient density to maintain transmission in an otherwise very low transmission setting. Malaria risk is well known and causes of malaria are well understood, but health risks posed by contracting malaria pale in comparison to the other hazards of logging and are therefore rarely a determinant of engaging in forest-based activities. Insecticide-treated bed nets and hammock nets are inadequate interventions to protect against biting vector exposure, given constraints on their use in the forest in this setting.

### Challenges and opportunities for malaria control and elimination.

Because of the inadequacy of currently available vector control tools to prevent forest malaria in this setting, interventions aim to interrupt malaria transmission by directly reducing the human parasite reservoir with some form of screening and treatment, mass drug administration, or active case detection. The enduring challenge with any of these approaches is the inverse relationship between risk and accessibility. The most mobile, high-risk forest-goers are also those who are least likely to be present in villages when a malaria control interventions take place, least likely to want to remain to complete a course of treatment, or to return for follow-up.

There is a clear need for long-term multi-sectoral approaches^32^ addressing the fundamental drivers of malaria in this region without which malaria elimination campaigns will continue to be limited. Nonetheless, the continued spread of multidrug-resistant *P. falciparum* parasites necessitates more urgent interventions over the next few years. There are a number of challenges involved in adapting strategies to prevent, detect, and treat malaria to the forest-going population’s realities. Existing tools including insecticide-treated clothing and personal topical repellents have been limited by insufficient and alternative uses,^33,34^ and there is an urgent need to develop new vector control tools specifically for use by forest workers.^35^ As groups of loggers reportedly congregate over relatively small distances in the forest and within flight ranges of *Anopheles* mosquitoes, spatial repellents and endectocides such as ivermectin may play a role but would need to be evaluated in this setting, and sufficient coverage levels will likely be difficult to achieve.^36^ The recent decline in organized logging by large migrant groups reduces the relevance of strategies that attempt to involve team leaders and brokers. However, in other settings, where logging is predominantly undertaken by larger organized migrant groups, this may still be a relevant and effective strategy. As most logging in this setting is now performed in smaller groups of local villagers who use many scattered entry and exit points to the forest, there are no obvious focal points where entry or exit screening and treatment could be setup. Again, in other settings where entry into the forest is limited to a few main entrances, this may be an effective strategy.

Despite transmission foci being located in the forest, the findings from this study suggest there are grounds for continuing to focus malaria control and elimination activities at village level in this setting. Community knowledge about the drivers of fluctuations in forest-going over short-time scales represents an under-recognized avenue for improving the targeting of forest-goers and the anticipation of and response to potential malaria outbreaks. Many residents (often including VMWs) are either forest-goers themselves or are embedded in familial and social networks in which information about forest resources and forest-going activities is openly discussed. The most appropriate intervention should be tailored according to risk and accessibility. Vigilant surveillance for malaria infections at village level, especially during local surges in forest-going, supported by sustained community engagement efforts may be more logistically feasible and effective than mass drug administrations or mass screenings, by taking advantage of time periods when forest-goers are in their home village to test and treat for malaria infections. Providing forest-goers with prophylactic treatment before forest-going may be effective, although this will require substantial strengthening of local health systems and crucially improved proactive communication about forest-going patterns between village residents, village leaders, and district and provincial health staff, despite the illegality of many forest-based activities. Where forest-goers are intending to stay in the forest for more than a week and presumptive treatment may be considered but given the emergence and spread of multidrug resistant malaria in this region, encouraging forest-goers to carry and self-treat with antimalarial medicines in the forest should be the last-resort option. One theoretically attractive alternative which has yet to be explored is the pre-exposure vaccination of forest-goers with the RTS, S vaccine.

Clearly, for any of these approaches, sustained community participation is critical,^37^ and efforts to build community participation will likely be strengthened if multi-sectoral approaches are developed.^32^ As observed elsewhere in the region, malaria risk in plantations can be managed through provision of insecticide-treated nets and regularization of sleeping spaces, as well as improved access to diagnosis and treatment.^38^ Cooperation between forest conservation, agricultural, and health-care sectors could plausibly lead to sustained co-benefits, improving livelihood opportunities, reducing deforestation, and enhancing feasibility of elimination of multidrug-resistant malaria.

## CONCLUSION

Focal malaria transmission among forest-goers in western Cambodia is likely to persist and challenge malaria elimination efforts. The target population is mobile, biologically and socioeconomically vulnerable, and dynamic. Efforts to reach them effectively require a flexible approach which builds on existing community knowledge and relationships. In the longer term, multi-sectoral approaches to address the fundamental drivers of malaria in this region, coupled with innovative and flexible malaria control strategies, may substantially increase the effectiveness of malaria elimination efforts.

## Footnotes

† Several hardwood trees native to the region are prized as luxury timbers, especially Siamese rosewood (*Dalbergia cochinchinensis* Pierre), known in the Khmer language as “Kranhoung.” Apart from rosewood, several other hardwood species are also collected, known in Khmer as “Beng” (*Afzelia xylocarpa*), “Thnong” (*Pterocarpus macrocarpus* Kurz) and “Neang Nuon” (*Dalbergia oliveri*). Some of these timbers are thought to be relatively plentiful than rosewood, but for now, these timbers sell at a lower price.
